# Missense Mutations of Human Hsp60: A Computational Analysis to Unveil Their Pathological Significance

**DOI:** 10.3389/fgene.2020.00969

**Published:** 2020-08-18

**Authors:** Alessandra Maria Vitale, Everly Conway de Macario, Riccardo Alessandro, Francesco Cappello, Alberto J. L. Macario, Antonella Marino Gammazza

**Affiliations:** ^1^Department of Biomedicine, Neuroscience and Advanced Diagnosis, Section of Human Anatomy, University of Palermo, Palermo, Italy; ^2^Euro-Mediterranean Institute of Science and Technology (IEMEST), Palermo, Italy; ^3^Department of Microbiology and Immunology, School of Medicine, University of Maryland at Baltimore-Institute of Marine and Environmental Technology (IMET), Baltimore, MD, United States

**Keywords:** Hsp60 gene variants, Hsp60 genetic chaperonopathies, chaperoning system, human genomes, underdiagnosed chaperonopathies

## Abstract

Two chaperonopathies have been linked to mutations in the human *hsp60* (*hHsp60*; *HSPD1*) gene, but other existing variants might cause diseases, even if there is no comprehensive information about this possibility. To fill this vacuum, which might be at the basis of misdiagnoses or simply ignorance of chaperonopathies in patients who would benefit by proper identification of their ailments, we searched the sequenced human genomes available in public databases to determine the range of missense mutations in the single *hsp60* gene. A total of 224 missense mutations were identified, including those already characterized. Detailed examination of these mutations was carried out to assess their possible impact on protein structure-function, considering: (a) the properties of individual amino acids; (b) the known functions of the amino acids in the human Hsp60 and/or in the highly similar bacterial ortholog GroEL; (c) the location of the mutant amino acids in the monomers and oligomers; and (d) structure-function relationships inferred from crystal structures. And we also applied a bioinformatics tool for predicting the impact of mutations on proteins. A portion of these genetic variants could have a deleterious impact on protein structure-function, but have not yet been associated with any pathology. Are these variants causing disease with mild clinical manifestations and are, therefore, being overlooked? Or are they causing overt disease, which is misdiagnosed? Our data indicate that more chaperonopathies might occur than is currently acknowledged and that awareness of chaperonopathies among medical personnel will increase their detection and improve patient management.

## Introduction

Hsp60, termed hHsp60 or HSPD1 in humans, is an essential chaperone typically residing in the mitochondria (mHsp60) and chloroplasts of eukaryotes and considered to have been derived from the ancestor of the bacterial counterpart currently known as GroEL. It belongs to the Group I chaperonins and, together with its co-chaperonin Hsp10 (mHsp10 in mitochondria), forms a macromolecular double-ring tetradecameric complex [mHsp60_14_-(mHsp10_7_)_2_], known as the football complex (Nisemblat et al., 2015), that assists the folding of proteins imported into the mitochondrial matrix and prevents their misfolding under stress condition (Hemmingsen et al., 1988; Cheng et al., 1989; Gupta, 1995). The double-ring tetradecameric complex is known for its intrinsic instability, since the purification attempts have shown that the mHsp60 is unstable and tends to dissociate into monomers at low temperatures and low protein concentrations (Viitanen et al., 1998; Vilasi et al., 2018). Moreover, in solution it exists in dynamic equilibrium between monomers, heptameric single rings and double-ring tetradecamers, and only the presence of both ATP and Hsp10 favors the formation of the functional football complex (Levy-Rimler et al., 2001). Not yet fully elucidated is also the mechanism of action, even if it likely involves different intermediates, including the ADP-bound half football complex and the ATP and ADP-bound football complexes (Gomez-Llorente et al., 2020).

While chaperones are classically considered cytoprotective, if abnormal, due to genetic or acquired modifications, can cause diseases, the chaperonopathies (Macario and Conway de Macario, 2005; Marino Gammazza et al., 2012; Scalia et al., 2019). For instance, two monogenic disorders have been associated with *HSPD1* gene missense mutations than can be deemed Hsp60-genetic chaperonopathies. They are: (1) a dominantly inherited form of spastic paraplegia – SPG13 (OMIM #605280), which is caused by the p.V98I and the p.Q461E missense mutations (Fontaine et al., 2000; Hansen et al., 2002, 2007); and (2) a recessively inherited hypo-myelinating leukodystrophy – HDL4 (OMIM #612233), caused by the p.D29G missense mutation (Magen et al., 2008; Kusk et al., 2016). While the clinical and pathological manifestations of these two neurological disorders are well characterized, less is known about the effects of the mutations on protein structure and function and their participation in the mechanisms underpinning the histopathological lesions observed in patients. It has been demonstrated that the mutations destabilize the oligomeric chaperonin complex and impair its ATPase and folding activities *in vitro* (Bross et al., 2008; Parnas et al., 2009). By a genetic complementation assay, it was shown that the mutants fail to replace the bacterial chaperonin GroEL (Hansen et al., 2002, 2007; Magen et al., 2008). It was also shown that the expression of the variants D29G and V98I alters mitochondria morphology, dynamics, and function, thereby causing mitochondrial dysfunction, cell death, and neurodegeneration (Miyamoto et al., 2015, 2016). However, the described instability of hHsp60 *in vitro* has hindered further investigations, and for this reason, the molecular mechanisms leading to disease-development associated with *HSPD1* mutations are not yet fully described.

Besides the three missense mutations of the *HSPD1* gene mentioned above, others have been reported (Bross and Fernandez-Guerra, 2016). It is, therefore, likely that the whole range of disorders associated with genetic variants of this gene is greater than is current established. It could very well be that there exist pathological conditions caused by hHsp60 variants that remain undiagnosed for various reasons such as, for instance, mild clinical manifestations or confusion with other diseases.

On this premise, we decided to investigate the range of hHsp60 genetic variants in the sequenced human genomes available to us and their potential pathological significance. We first identified, using available databases, all the known *HSPD1* gene missense mutations, finding 224 variants of which 27 have been already associated with a pathological condition, but with an uncertain clinical significance, except for the three known mutations mentioned above. We focused on 15 variants that we considered to span the whole range of possible impacts on the functionality of the chaperonin, i.e., from neutral to highly damaging. We sought to predict the impact of sequence changes on protein structure and biological function, and their possible disease-association considering: (a) the properties of individual amino acids; (b) the function of these amino acids in the human chaperonin, if known, or that of the counterparts in the highly similar bacterial ortholog GroEL; (c) the location of the mutant amino acids in the monomers and oligomers; and (d) structure-function relationships inferred from crystal structures. And we also applied a bioinformatics tool designed to predict the possible impact of amino acid substitutions on the stability and function of human proteins.

We chose the mutations involving the residues Ala34, Asp35, Glu129, Lys133, Ly473, and Ser488, because these amino acids are involved in inter-ring contacts, as shown by crystal structures (Nisemblat et al., 2015; Gomez-Llorente et al., 2020). For the other chosen missense mutations, which involve the residues Met55, Arg221, Tyr223, Asn265, Val287, Glu328, Asp504, Ala505, and Met506 with an unknown function in human chaperonin, we based our analysis on their bacterial counterparts, that maintain the same identity, and/or localization in three-dimensional structure, and have a known role.

## Materials and Methods

### Identification of Human *HSPD1* Gene Missense Mutations

A comprehensive search of known human *HSPD1* missense mutations was carried out, and the data were tabulated, using information from the literature and three databases: the Genome Aggregation Database (gnomAD^1^), which includes exome and genome sequencing data from a variety of large-scale sequencing projects; the NHLBI GO Exome Sequencing Project [Exome Variant Server, NHLBI Exome Sequencing Project (ESP), Seattle, WA^2^ ], whose objectives are to reveal novel genes implicated in heart, lung, and blood pathological conditions, using next-generation sequencing of human exomes; and the ClinVar database^3^, curated by the National Center for Biotechnology Information (NCBI) that collects data on genome variants and their impact on human health.

### Computational Predictions of the Effects of Selected Missense Mutations

In addition to the exhaustive comparative analysis of structural details and amino acid properties, we used the bioinformatics tool PolyPhen-2 (Polymorphism Phenotyping v2^4^), to assess the effects of hHsp60 gene missense mutations on protein structure/function and possible pathogenicity. PolyPhen-2 predicts the impact of amino acid substitutions on the stability and function of human proteins using structural and comparative evolutionary parameters and providing three prediction outputs, i.e., “probably damaging”, “possibly damaging”, and “benign” (Adzhubei et al., 2013).

### Bacterial GroEL and hHsp60 Sequences Alignment

The amino acid sequences of bacterial GroEL and hHsp60 were obtained from the NCBI Protein database (NCBI Reference Sequences: NP_418567.1 and NP_002147.2, respectively), following the cross references found in the dedicated webpages of the UniprotKB database (P0A6F5 for GroEL and P10809 for hHsp60).

The pairwise sequence alignment was performed using the BLASTp suite of the BLAST alignment tool^5^, loading as query the GroEL sequence and as subject the hHsp60 sequence, and using the algorithm parameters given by default.

### Bacterial GroEL and hHsp60 Structure Comparison

The GroEL and hHsp60 proteins’ monomeric structures were built using a fully automated protein structure homology-modeling server named SWISS-MODEL^6^ (Arnold et al., 2006; Guex et al., 2009; Waterhouse et al., 2018). Among the obtained model results, for GroEL was chosen the Model 01, built using as template the chain B of the crystal structure of the football-shaped GroEL-GroES2-(ADPBeFx)14 complex, deposited into the RCSB PDB^7^ under the PDB ID 4PKO (Fei et al., 2014). For hHsp60 was chosen the Model 01, built using as template the chain J of the crystal structure of the ADP:BeF3 bound mHsp60–mHsp10 football complex, deposited into the RCSB PDB under the PDB ID 6HT7 (Gomez-Llorente et al., 2020).

Both models were visualized with PyMOL Molecular Graphics System, Version 2.0 Schrödinger, LLC^8^. The same program was used to model-visualize the GroEL and hHsp60 single monomers superimposition, as well as the localization of some mutant residues.

## Results

### GroEL and hHsp60 Primary and Tertiary Structures

**FIGURE 1:**
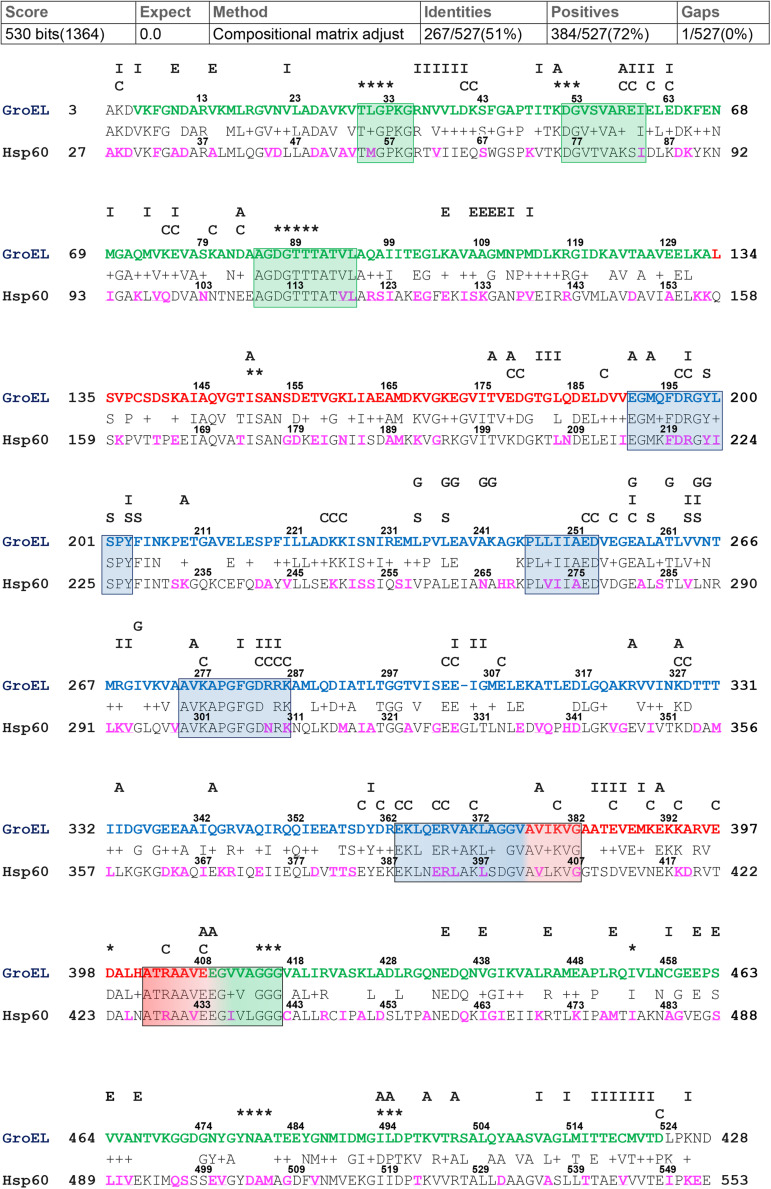
GroEL and hHsp60 amino acid sequences alignment. The alignment was obtained using BLASTp and the GroEL (NP_418567.1) and hHsp60 (NP_002147.2) sequences. **Top**, GroEL sequence: green letters correspond to the equatorial domain (residues 6-133 and 409-523); red letters correspond to the intermediated domain (residues 134-190 and 377-408); blue letters correspond to the apical domain (residues 191-376) (Xu et al., 1997). Highly conserved peptides are boxed. The letters and asterisks above the GroEL sequence indicate the known function of the residues underneath: I, intra-ring contacts; E, inter-ring contacts; A, intra-monomer contacts; C, charged residues exposed to the central cavity in the *cis* conformation; S, substrate binding; G, GroES binding; asterisks, ATP/ADP and Mg^2+^ binding (Braig et al., 1994; Fenton et al., 1994; Boisvert et al., 1996; Xu et al., 1997; Brocchieri and Karlin, 2000; Wang and Boisvert, 2003). **Bottom**, hHsp60 sequence: The amino acids found mutated are in purple font.

For the alignment of GroEL and hHsp60 sequences, the BLASTp output reported 51% identity and 72% positivity (residues which differ between the two compared sequences but maintain the same chemical properties and are considered as conservative substitutions), and a single gap located at residue number 305 of the bacterial chaperonin (Figure 1). As shown, the alignment of the human chaperonin starts at alanine 27 and ends with the residue 553. Therefore, the similarity of the hHsp60 with the bacterial protein excludes the mitochondrial import signal (MIS), i.e., the N-terminal amino acids 1 to 26, typical of the human molecule before entering the mitochondria.

**FIGURE 2:**
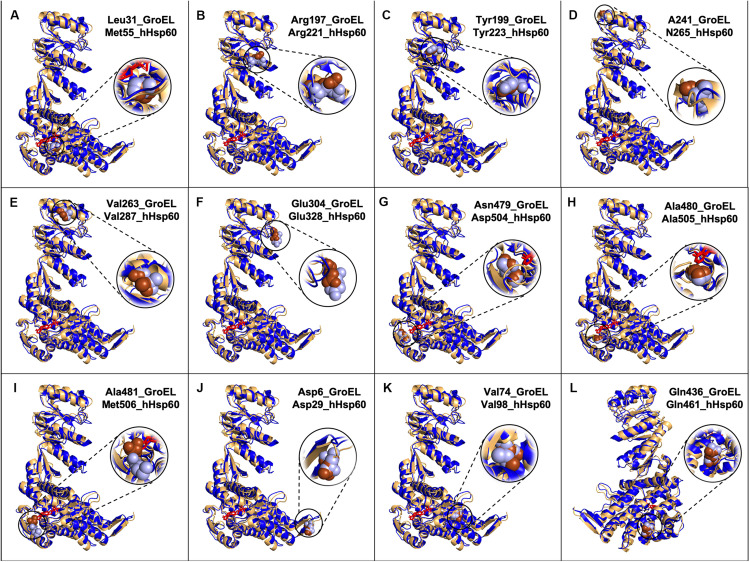
Models of GroEL (light brown) and hHsp60 (blue) wild type monomers superimposed and location of some of the amino acids found mutated in hHsp60 (light blue spheres), and their corresponding amino acids in GroEL (brown spheres). For both molecules, the nucleotide is colored in red. Models were built using SWISS-MODEL and visualized with PyMOL. **(A)** GroEL leucine 31 and hHsp60 methionine 55; **(B)** GroEL arginine 197 and hHsp60 arginine 221; **(C)** GroEL tyrosine 199 and hHsp60 tyrosine 223; **(D)** GroEL alanine 241 and hHsp60 asparagine 265; **(E)** GroEL valine 263 and hHsp60 valine 287; **(F)** GroEL glutamic acid 304 and hHsp60 glutamic acid 328; **(G)** GroEL asparagine 479 and hHsp60 aspartic acid 504; **(H)** GroEL alanine 480 and hHsp60 alanine 505; **(I)** GroEL alanine 581 and hHsp60 methionine 506; **(J)** GroEL aspartic acid 5 and hHsp60 aspartic acid 29; **(K)** GroEL valine 74 and hHsp60 valine 98; **(L)** GroEL glutamine 436 and hHsp60 glutamine 461 (the molecules were rotated 200 degrees around the *y*-axis compared to the other figures).

The great similarity between the mHsp60 and GroEL monomers was evident also when three-dimensional models were compared (Figure 2).

### Human *HSPD1* Gene Missense Mutations

A total of 224 missense mutations of the *HSPD1* gene were found (Supplementary Table S1), which would cause specific amino acid replacements in the encoded protein sequence. Many of these missense mutations appeared in all the consulted databases, while others were reported only in one of them. For instance, GnomAD lists 208 missense variations for the protein product of the canonical HSPD1 transcript; NHLBI ESP reports 23 missense mutations, also for the canonical HSPD1 transcript; and ClinVar lists 29 missense mutations. In addition, as shown in Supplementary Table S1, some amino acids may be involved in two or three different mutations.

Among the 29 missense mutations recorded in the ClinVar database, there are the two missense mutations associated with the development of neurodegenerative diseases mentioned in the introduction, i.e., p.V98I and p.D29G. Moreover, other 24 mutations are currently associated with the broad and heterogenous group of spastic paraplegias, even if for most of them the clinical significance is uncertain or conflicting (for details see the corresponding ClinVar webpages). The amino acids involved in missense mutations and belonging to the sequence that can be aligned with GroEL were visualized in proteins three-dimensional models (data not shown for all the missense mutations), and highlighted in purple in the subject sequence (hHsp60 sequence) of the performed pairwise alignment (Figure 1). Only 169 mutations occur at positions that align with GroEL, while the remaining occur in the MIS or the C-terminal portion of hHsp60, both without match in GroEL.

#### Missense Mutations Occurring in Residues Involved in Inter-Ring Contacts

Five key inter-ring contacts in hHsp60 revealed by crystallography (Nisemblat et al., 2015; Gomez-Llorente et al., 2020) involve amino acids that have been found mutated. The mutations found are A34V, D35G, E129K, K133E, K473Q, and S488T. Only E129K is already associated with spastic paraplegia, in accordance with the PolyPhen-2 prediction result, giving a possibly damaging effect. On the contrary, the others are not associated with any disease and PolyPhen-2 predicted a possible damaging effect only for S488T (Table 1 and Supplementary Table S1).

**TABLE 1:** Illustrative examples of human Hsp60 missense mutations in sequenced genomes.

Mutation	Disease	Source	Pathogenicity^1^	Pathogenicity prediction by PolyPhen-2
p.D29G	Hypomyelinating leukodystrophy	(Magen et al., 2008; Parnas et al., 2009; Bross and Fernandez-Guerra, 2016); ClinVar	Proven	Benign
p.A34V	N. R.^2^	gnomAD	Unlikely	Benign
p.D35G	N. R.	gnomAD	Unlikely	Benign
p.M55L	N. R.	gnomAD	Unlikely	Benign
p.V98I	Spastic paraplegia 13 (SPG13)	(Fontaine et al., 2000; Hansen et al., 2002; Bross et al., 2008; Bross and Fernandez-Guerra, 2016); ClinVar	Proven	Possibly damaging
p.E129K	Spastic paraplegia	ClinVar; gnomAD; NHLBI ESP	Likely	Possibly damaging
p.K133E	N. R.	ClinVar	Likely	Benign
p.R221Q	Hereditary spastic paraplegia 13	ClinVar; gnomAD; NHBLI ESP	Likely	Benign
p.Y223C	N. R.	gnomAD	Likely	Probably damaging
p.N265S	Hereditary spastic paraplegia 13	(Bross and Fernandez-Guerra, 2016); ClinVar; gnomAD; NHLBI ESP	Likely	Possibly damaging
p.V287I	Hereditary spastic paraplegia 13	ClinVar	Likely	Benign
p.E328V	N. R.	gnomAD	Unlikely	Possibly damaging
p.Q461E	Autosomal dominant spastic paraplegia (SPG13)	(Hansen et al., 2007; Bross and Fernandez-Guerra, 2016)	Proven	Probably damaging
p.K473Q	N. R.	gnomAD	Likely	Benign
p.S488T	N. R.	gnomAD	Likely	Possibly damaging
p.D504N	N. R.	gnomAD	Unlikely	Benign
p. A505T	N. R.	gnomAD	Unlikely	Benign
p.M506V	N. R.	gnomAD	Unlikely	Benign

*^1^Assessed by the study of the possible consequence of mutations on hHsp60 structure related to the function and chemical-physical properties of the substituted amino acids (see text). ^2^N.R., none reported.*

#### Missense Mutations Occurring in Conserved Residues

The research conducted in the mentioned databases revealed that other potentially interesting missense mutations involve residues (Met55, Arg221, Tyr223, Asn265, Val287, Glu328, Asp504, Ala505, and Met506) with an unknown function in the human chaperonin. These residues maintain the same identity and/or localization in GroEL (Figures 1, 2A–I). The mutations involving these residues are M55L, R221Q, Y223C, N265S, V287I, E328V, D504N, A505T, and M506V.

R221Q, N265S, and V287I are associated with spastic paraplegia. However, the prediction of pathogenicity by the bioinformatics tool was often contradictory with the disease-association for these mutations. The remaining mutations are not disease associated, and PolyPhen-2 predicted a possible damaging effect only for Y233C and E328V (Table 1 and Supplementary Table S1).

#### Missense Mutations With Proved Disease Association

D29G, V98I, and Q461E are hHsp60 missense mutations associated with hypomyelinating leukodystrophy and hereditary spastic paraplegia 13. These residues maintain the same identity (Figure 1) and localization (Figures 2J–L) in GroEL. However, the functions of the corresponding amino acids in the bacterial chaperonin, i.e., Asp5, Val74, and Gln436, have not yet been defined (Figure 1). Moreover, the prediction tool of pathogenicity produced divergent results such as benign for D29G, possibly and probably damaging for V98I and Q461E, respectively (Table 1).

## Discussion

*HSPD1* missense mutations can cause Hsp60-genetic chaperonopathies as indicated by the fact that among the identified 224 variants, 27 were associated with a pathological condition. These are spastic paraplegias and hypomyelinating leukodystrophy, as mentioned earlier.

The tight association between a defective hHsp60 and neurodegeneration is due to the great sensitivity of neuronal cells to mitochondrial dysfunction (Bross et al., 2013).

However, despite the clear association between Hsp60 functionality, mitochondrial integrity, and neuronal tissue homeostasis, the role of the chaperonin, when abnormal, as etiologic-pathogenic factor in the development of neurodegenerative diseases is still not widely appreciated. This most likely leads to misinterpretation of clinical-pathological findings and erroneous diagnoses of a range of rare neurological disorders. In fact, except for the three well-known pathogenic mutants, there are no data on the potential pathological significance pertaining to the other variants.

The reasons for this scarcity of information are unclear but lack of physician preparedness to identify chaperonopathies may be one, and the other could be the difficulty in the isolation and purification of the mitochondrial chaperonin, whose unstable structure can easily be affected by external factors, hindering investigations on its native structure, function and mechanisms of action (Viitanen et al., 1998; Levy-Rimler et al., 2001; Vilasi et al., 2018). Consequently, a considerable part of our knowledge about hHsp60 comes from the extensive studies on the bacterial GroEL that was often used as model given its sequence similarity with the human counterpart together with its almost identical three-dimensional organization.

These similarities strongly support the notion that also the functions of the amino acids located at equivalent positions in the two sequences are conserved. Noteworthy is the fact that the most conserved amino acids are those responsible for essential functions such as nucleotide binding, intra- or inter-rings contacts, co-chaperonin interaction, and substrate binding.

In our work, detailed examination of 15 mutations was carried out to assess their possible impact on hHsp60 structure-function. The data were further enriched with the results obtained with PolyPhen-2, a bioinformatics prediction tool. In addition to the pathogenicity prediction, PolyPhen-2 also provides a multiple sequence alignment showing conservation of the residues involved in the considered mutation in many organisms. This type of information is of interest to appreciate the evolutionary-weighted conservation of these residues. However, in this work we focused only on the similarity between hHsp60 and GroEL.

The mutations A34V, D35G, E129K, K133E, K473Q, and S488T occur in residues involved in the inter-ring contacts, and for this reason, they could compromise the formation and the stability of the double-ring tetradecamer, at least in the football complex, impairing the chaperoning function. Regarding the residue at position 34, involved in a symmetric hydrophobic interaction with the same residue from the opposite ring (Nisemblat et al., 2015) both the original amino acid (alanine) and the substituted one (valine) are hydrophobic, so it is likely that valine could maintain the described contact, without altering protein structure/function. This coincides with the bioinformatics result that predicted a benign effect of this substitution (Table 1). The residue at position 35 is an aspartic acid involved in a symmetric hydrogen bond with the same residue from the opposite ring (Nisemblat et al., 2015). Asp35 is a polar amino acid, while the substituted one (glycine) is hydrophobic and could prevent the formation of the mentioned inter-ring bond, destabilizing the double-ring tetrameric complex. However, also in this case, the bioinformatics tool predicted a benign effect for the substitution on protein structure/function (Table 1). In accordance with the predictions none of these two missense mutations has yet been found associated with a pathological condition (Table 1 and Supplementary Table S1).

Glu129 and Lys133 of hHsp60 full-length, corresponding to Glu103 and Lys107 in mHsp60, are linked by a salt bridge (Nisemblat et al., 2015; Gomez-Llorente et al., 2020). Both these residues were found replaced by a lysine and a glutamic acid, respectively (Table 1 and Supplementary Table S1), and both these replacements could prevent the formation of the described salt bridge, altering the stability of the tetradecamer. However, despite these hypothetical consequences, only the E129K missense mutation was associated with the development of spastic paraplegia (Table 1 and Supplementary Table S1), which is in accordance with the prediction by PolyPhen-2 that classified it as possibly damaging (Table 1). Conversely, for the K133E missense mutation, there is no reported disease-association (Table 1 and Supplementary Table S1), which is in accordance with the bioinformatics prediction that classified it as benign. Likewise, the missense mutation involving Lys473 of hHsp60 full-length, corresponding to Lys447 of mHsp60, and replacing it with a glutamine (Table 1 and Supplementary Table S1) could compromise the salt bridge with Glu486 (Glu460 in mHsp60), revealed by crystallography (Nisemblat et al., 2015). The bioinformatics tool predicted a benign effect on protein structure/function, which coincides with the fact that there is no reported pathological condition attributed to this mutation (Table 1 and Supplementary Table S1).

Also S488, corresponding to S462 in mHsp60, which has been recently reported to be involved in inter-ring contacts (Gomez-Llorente et al., 2020), was found to be replaced by a threonine (Table 1 and Supplementary Table S1). The important role of this amino acid suggests a potential deleterious effect of its substitution. The bioinformatics tool predicted a possible negative effect for this amino acid replacement on protein structure/function (Table 1). However, no pathological condition has yet been associated with this missense mutation (Table 1 and Supplementary Table S1). The reason could be the chemical similarity of the involved residues which are both neutral polar amino acids. Thus, it is likely that threonine keeps on allowing the formation of the inter-ring contacts and, at least, the formation of the double-ring complex.

Other potentially interesting missense mutations involve residues with an unknown function in human chaperonin. For this reason, we analyzed them considering the bacterial counterpart, in which these residues maintain the same identity and/or localization in the three-dimensional structure and have a known role.

Met55 sits in the nucleotide binding pocket, as inferred from the analysis of the recent solved crystal structure of the ADP:BeF3-bound (ATP ground state) wild-type mHsp60_14_–(mHsp10_7_)_2_ football complex (Gomez-Llorente et al., 2020). Met55 corresponds to GroEL Leu31 that is known to be involved in nucleotide binding (Xu et al., 1997; Brocchieri and Karlin, 2000; Wang and Boisvert, 2003). So, even if the residue is different, it maintains the same localization and, probably, the same function in both molecules. In M55L missense mutation Met55 was replaced by a leucine (Table 1 and Supplementary Table S1), also a non-polar amino acid but with a different side chain. This difference may have an impact on the hHsp60 structure and function. Interesting, this substitution would make the residue at position 55 of hHsp60 identical to the corresponding amino acid in the GroEL sequence and that it is involved in nucleotide binding, which could explain why this mutation has not yet been found implicated in a pathological condition (Table 1 and Supplementary Table S1). Also the prediction tool gave a comparable result, with a predicted benign impact on protein structure/function (Table 1).

Another interesting residue is Arg221, a basic polar amino acid located within a highly conserved peptide and aligned with the bacterial Arg197, which is one of the polar amino acids exposed to the central cavity, and is involved in a charged intra-ring contact (Figure 1) (Braig et al., 1994; Boisvert et al., 1996; Xu et al., 1997; Brocchieri and Karlin, 2000). The pairwise alignment and the three-dimensional structures superimposition (Figures 1, 2B) show that the residue maintains not only the same identity but also the same position in the bacterial and human chaperonins, possibly also conserving its function. Arg221 can be replaced by a glutamine, a polar, but non-basic amino acid, and this substitution was found associated with hereditary spastic paraplegia 13. However, no further data regarding this disease-association are available in the current literature, and the prediction result showed disagreement, giving a benign prediction.

Also Tyr223, a neutral polar amino acid with an aromatic side chain, is localized in the apical domain in the same highly conserved sequence containing Arg221 and corresponds to bacterial Tyr199 that is involved in substrate binding (Figure 1) (Fenton et al., 1994; Xu et al., 1997; Brocchieri and Karlin, 2000). Thus, in the human and bacterial chaperonins, the residue maintains the same identity and localization (Figure 2C), suggesting conservation of functionality. In the mutation Y223C, Tyr223 was found replaced by a cysteine, a hydrophobic amino acid with a thiol group in its side chain, instead of the aromatic ring typical of a tyrosine. Since the two amino acids have different chemical properties and size, we can hypothesize that this replacement is non-functional. In accordance with our hypothesis, the used prediction tool gave a high score of damaging effect on protein structure/function (Table 1). Nevertheless, no pathological condition has yet been reported associated with this missense mutation (Table 1 and Supplementary Table S1).

Human Asn265 is in the apical domain within the H helix that, together with the I helix, forms the interface with the Hsp10 mobile loop. In the linear sequence alignment, this residue corresponds to bacterial Ala241 (Figure 1), which is involved in the interaction with GroES (Brocchieri and Karlin, 2000), and, as shown in Figure 2D, also their localizations are the same. Hence, the function could be maintained. In fact, we searched for Asn265 in the last solved crystal structure of the ADP:BeF3-bound (ATP ground state) wild-type mHsp60_14_–(mHsp10_7_)_2_ football complex (Gomez-Llorente et al., 2020), available in RCSB PBD, and we found that this residue interacts with a residue belonging to the mobile loop of Hsp10 (M32) (data not shown). In the mutation N265S, Asn265 was found replaced by serine, which like asparagine is a neutral amino acid, but with a different side chain (Table 1 and Supplementary Table S1). Therefore, it is possible that the mutation N265S destabilizes the interaction of hHsp60 with the Hsp10, leading to a pathological condition. In accordance with this hypothesis, also the prediction tool predicted possible damaging/deleterious effect on protein structure/function (Table 1). As reported in the ClinVar database, this missense mutation was found associated with hereditary spastic paraplegia 13 (Table 1 and Supplementary Table S1). However, no other information about this pathologic entity can be found in the literature, and interpretations about its pathogenicity remain conflictual.

The missense mutation involving the residue Val287 (V287I) is associated with hereditary spastic paraplegia 13, as reported in the ClinVar database (Table 1 and Supplementary Table S1). Also, in this case, to clarify how this replacement could affect protein structure and function leading to disease development, we compared the human chaperonin with its bacterial counterpart since the function of Val287 is not known. This residue is located in the apical domain within the I helix and has the same identity (Figure 1) and localization (Figure 2E) of the corresponding residue in GroEL (Val263), which is involved in substrate binding and in contacts between neighboring monomers of the same ring (Fenton et al., 1994; Brocchieri and Karlin, 2000). Moreover, Val263 is located near other residues involved in the interaction with the co-chaperonin (Figure 1). Thus, it could maintain the same functions. Replacement of Val287 with isoleucine, also a non-polar aliphatic amino acid but with a different molecular mass, could be pathogenic (Table 1 and Supplementary Table S1). It could impair the formation and the stability of the Hsp60 double ring tetradecamer, and its interaction with the co-chaperonin and the substrate by creating a steric bulk. However, there is still uncertainty in the interpretation of the pathogenicity of this mutation, since the result given by the used prediction tool classifies this substitution as benign (Table 1).

Another important function is the interaction among neighboring monomers that ensures the stability of both single and double-ring complexes. For example, Glu304 in GroEL is a residue involved in this type of interaction and in the substrate binding (Brocchieri and Karlin, 2000) and corresponds to Glu328 in hHsp60, a negatively charged residue sited in the apical domain of the human chaperonin (Figures 1, 2F). In the mutation E328V, Glu328 was found replaced by a valine, a hydrophobic amino acid that cannot interact with a folding substrate but could still be involved in intra-ring contacts. The prediction obtained with the used bioinformatics tool indicated a possibly damaging effect of this substitution on protein structure/function (Table 1). Nevertheless, this replacement has not been associated with a pathological condition (Table 1 and Supplementary Table S1).

The residues Asp504, Ala505, and Met506 are located in the nucleotide-binding pocket of hHsp60, as inferred from the solved crystal structures (Nisemblat et al., 2015; Gomez-Llorente et al., 2020). The corresponding amino acids in GroEL, i.e., Asn479, Ala480, and Ala481, involved in nucleotide binding (Boisvert et al., 1996; Xu et al., 1997; Brocchieri and Karlin, 2000), have different identities, but maintain the same location in proteins three-dimensional structures (Figures 1, 2G–I). Thus, it is likely that the function could be maintained. In human chaperonin, these three residues were found affected by missense mutations (D504N, A505T, M506V) but, despite their presumably important function, none of these variants seemed to cause a pathological condition (Table 1 and Supplementary Table S1), and the automatic prediction tool result agreed with this assumption (Table 1).

With regard to the three missense mutations in hHsp60 clearly associated with the development of neurodegenerative disease, i.e. D29G, V98I, and Q461E, we would like to report a brief analysis of their intrinsic characteristics in relation also to GroEL. These residues maintain the same identity and localization in GroEL. However, the functions of the corresponding amino acids in GroEL, i.e., Asp5, Val74, and Gln436, have not yet been defined (Figure 1). Asp29, a polar, negatively charged amino acid, is replaced by a glycine, which is a hydrophobic amino acid; Val98, a non-polar aliphatic amino acid, is replaced by an isoleucine, which is a hydrophobic amino acid that has a different molecular mass; Gln461, a polar uncharged amino acid, is replaced by a glutamic acid, which is the corresponding negatively charged acidic form. It can, therefore, be assumed that the differences of their chemical properties are responsible for the observed oligomeric complex instability and the alteration of protein functions (ATPase and folding activities), leading to defects in nervous and muscular tissue homeostasis and disease development.

A range of variants of the hHsp60 genes are present in the human genomes sequenced thus far. These variants can be sorted into three groups considering the probability of pathogenicity: mutants unlikely to have an impact on the structure-function of hHsp60 and, therefore, unlikely to be pathogenic, such as M55L and A34V; mutants bound to be pathogenic because of a predictable damaging impact on structure-function of hHsp60 that have already been found associated with disease, i.e. D29G, V98I and Q461E; and mutants with potential for disrupting structure-function of hHsp60, and so likely to be pathogenic. These latter include mutations such as E129K, K133E, N265S, and V287I which have been either associated or not with disease, but with an uncertain/conflicting interpretation. In this regard, the results obtained with the prediction tool were inconclusive since in many cases they disagreed with the impact of an amino acid substitution on protein structure/function and, consequently with the disease-association. Then, the automatic prediction results must be considered with caution.

So far, only a few mutants have been directly associated with pathologies, even if many others affect sites in the chaperonin molecule that are critical for proper folding, oligomerization, and chaperoning function and could, therefore, cause disease. Two questions then arise: are there Hsp60 genetic chaperonopathies with mild clinical manifestations that go unnoticed in routine clinical examinations? and/or are there chaperonopathies showing clear clinical symptoms that are misdiagnosed? Both situations are possible, with some degree of probability, because chaperonopathies are not yet taught in medical schools in general and can, therefore, easily be overlooked in practice.

It is hoped that specific preparation of medical professionals and their awareness of the existence of chaperonopathies will provide answers to the above questions. Furthermore, discovery of chaperonopathies and assessment of their true incidence starting at birth, or even prenatally, will contribute toward a better understanding of these sometimes-serious disorders and to the developing of specific therapies.

## Data Availability Statement

The datasets presented in this study can be found in online repositories. The names of the repository/repositories and accession number(s) can be found below: https://www.ncbi.nlm.nih.gov/, NP_002147.2; https://www.ncbi.nlm.nih.gov/, NP_418567.1; http://www.wwpdb.org/, 4PJ1; http://www.wwpdb.org/, 3WVL; https://www.ncbi.nlm.nih.gov/clinvar/, HSDP1; https://gnomad.broadinstitute.org/about, HSPD1; and https://evs.gs.washington.edu/EVS/, HSPD1.

## Author Contributions

AV gathered the data, materials, and references, wrote the manuscript drafts, and prepared the figures. EC, RA, and FC critically examined the data and references and contributed to the writing. AM critically examined the data and references and contributed to the discussion and the writing. AMG conceived the study, critically examined the data and materials, and contributed to the discussion and the writing. All authors contributed to the article and approved the submitted version.

## Conflict of Interest

The authors declare that the research was conducted in the absence of any commercial or financial relationships that could be construed as a potential conflict of interest.

## Footnotes

1 https://gnomad.broadinstitute.org/about

2 https://evs.gs.washington.edu/EVS/

3 https://www.ncbi.nlm.nih.gov/clinvar/

4 http://genetics.bwh.harvard.edu/pph2/index.shtml

5 https://blast.ncbi.nlm.nih.gov/Blast.cgi

6 https://swissmodel.expasy.org/

7 http://www.rcsb.org/

8 https://pymol.org/2/
