# Supplementary material for: Missense Mutations of Human Hsp60: A Computational Analysis to Unveil Their Pathological Significance
Source: Front Genet. 2020 Aug 18;11:969. doi: 10.3389/fgene.2020.00969 (PMC7461820; doi:10.3389/fgene.2020.00969)
Supplement: Supplementary file 1 [file Table_1.DOCX]

**Table S1.** Human Hsp60 missense mutations^1^

| **Mutation** | **Disease** | **Source** |
| --- | --- | --- |
| p.Arg3Leu | None reported | gnomAD |
| p.Arg3Trp | None reported | gnomAD |
| p.Arg9Gly | None reported | NHBLI ESP |
| p.Arg9Leu | None reported | NHBLI ESP |
| p.Gln10His | Spastic paraplegia | gnomAD  ClinVar |
| p.Gln10His | None reported | gnomAD |
| p.Pro13Arg | None reported | gnomAD |
| p.Pro13Gln | None reported | gnomAD |
| p.Pro13Leu | None reported | gnomAD  NHBLI ESP |
| p.Val14Ala | None reported | gnomAD |
| p.Ser15Ala | None reported | gnomAD |
| p.Arg16Lys | None reported | gnomAD |
| p.Val17Ala | None reported | gnomAD |
| p.Leu18Val | None reported | gnomAD |
| p.Pro20Leu | None reported | gnomAD |
| p.His21Asp | None reported | gnomAD  NHBLI ESP |
| p.His21Pro | None reported | gnomAD |
| p.Ala27Gly | None reported | gnomAD  NHBLI ESP |
| p.Lys28Arg | None reported | gnomAD |
| p.Asp29Gly | Hypomyelinating leukodystrophy | (Magen et al., 2008; Bross and Fernandez-Guerra, 2016; Kusk et al., 2016)  ClinVar |
| p.Phe32Cys | None reported | gnomAD |
| p.Ala34Val | None reported | gnomAD |
| p.Asp35Gly | None reported | gnomAD |
| p.Ala38Asp | None reported | gnomAD |
| p.Val44Ile | Spastic paraplegia | ClinVar |
| p.Asp45Ala | None reported | gnomAD |
| p.Asp49His | Hereditary spastic paraplegia 13 | ClinVar |
| p.Ala50Ser | None reported | gnomAD |
| p.Ala52Asp | None reported | gnomAD |
| p.Val53Phe | None reported | gnomAD |
| p.Met55Leu | None reported | gnomAD |
| p.Val62Ala | None reported | gnomAD |
| p.Ser67Gly | None reported | gnomAD |
| p.Lys72Arg | None reported | gnomAD |
| p.Ile84Val | None reported | gnomAD |
| p.Asp88Glu | None reported | gnomAD |
| p.Lys89Thr | None reported | gnomAD |
| p.Ile93Met | None reported | gnomAD |
| p.Ile93Val | None reported | gnomAD  NHBLI ESP |
| p.Lys96Asn | None reported | gnomAD |
| p.Val98Ile | Hereditary spastic paraplegia 13 | (Fontaine et al., 2000; Hansen et al., 2002; Bross et al., 2008; Bross and Fernandez-Guerra, 2016)  ClinVar |
| p.Gln99Glu | None reported | gnomAD |
| p.Asn103Ser | None reported | gnomAD |
| p.Val118Ile | None reported | gnomAD |
| p.Val118Leu | None reported | gnomAD |
| p.Leu119Val | None reported | ClinVar |
| p.Arg121His | None reported | gnomAD |
| p.Ser122Cys | None reported | gnomAD |
| p.Ile123Val | None reported | gnomAD |
| p.Glu126Asp | None reported | gnomAD |
| p.Gly127Ala | None reported | gnomAD |
| p.Gly127Asp | None reported | gnomAD |
| p.Glu129Lys | Spastic paraplegia | ClinVar  gnomAD  NHBLI ESP |
| p.Ile131Thr | None reported | gnomAD |
| p.Ser132Asn | None reported | gnomAD |
| p.Lys133Glu | None reported | ClinVar |
| p.Pro137Ser | None reported | gnomAD |
| p.Val138Gly | None reported | gnomAD |
| p.Arg142Lys | Spastic Paraplegia | (Bross and Fernandez-Guerra, 2016)  ClinVar  gnomAD  NHBLI ESP |
| p.Asp149Asn | None reported | gnomAD |
| p.Asp149His | None reported | gnomAD |
| p.Ala153Ser | None reported | gnomAD |
| p.Lys156Arg | None reported | (Bross and Fernandez-Guerra, 2016)  gnomAD |
| p.Lys157Arg | Not provided | gnomAD |
| p.Lys160Glu | Spastic Paraplegia | ClinVar |
| p.Thr164Ile | None reported | gnomAD |
| p.Glu166Asp | None reported | gnomAD |
| p.Thr173Met | None reported | gnomAD |
| p.Gly178Arg | None reported | gnomAD |
| p.Asp179Asn | None reported | gnomAD  NHBLI ESP |
| p.Glu181Val | None reported | gnomAD  NHBLI ESP |
| p.Ile182Thr | None reported | gnomAD |
| p.Asn184Ser | Hereditary Spastic Paraplegia | (Hansen et al., 2002; Bross and Fernandez-Guerra, 2016)  ClinVar  gnomAD  NHBLI ESP |
| p.Ile186Leu | None reported | gnomAD |
| p.Ala189Val | None reported | gnomAD |
| p.Met190Val | Spastic Paraplegia | ClinVar  gnomAD |
| p.Lys192Glu | Spastic Paraplegia | ClinVar  gnomAD |
| p.Gly194Glu | Hereditary spastic paraplegia 13 | ClinVar |
| p.Leu207Val | None reported | gnomAD |
| p.Asn208Thr | None reported | gnomAD |
| p.Ile214Thr | None reported | gnomAD |
| p.Phe219Val | None reported | gnomAD |
| p.Asp220Glu | None reported | gnomAD |
| p.Arg221Gln | Hereditary spastic paraplegia 13 | ClinVar  gnomAD  NHBLI ESP |
| p.Tyr223Cys | None reported | gnomAD |
| p.Ile224Thr | None reported | gnomAD |
| p.Ser232Pro | None reported | gnomAD |
| p.Lys233Gln | Spastic Paraplegia | ClinVar |
| p.Asp241Asn | None reported | gnomAD |
| p.Ala242Thr | None reported | gnomAD |
| p.Ala242Val | None reported | gnomAD |
| p.Val244Ile | None reported | gnomAD |
| p.Lys249Arg | None reported | gnomAD |
| p.Ile251Phe | None reported | gnomAD |
| p.Ser252Ala | None reported | gnomAD  NHBLI ESP |
| p.Ser253Asn | None reported | gnomAD |
| p.Ser253Thr | None reported | gnomAD |
| p.Ser256Ala | None reported | gnomAD |
| p.Ile257Val | None reported | gnomAD |
| p.Asn265Ser | Hereditary spastic paraplegia 13 | (Bross and Fernandez-Guerra, 2016)  ClinVar  gnomAD  NHBLI ESP |
| p.His267Tyr | None reported | gnomAD  NHBLI ESP |
| p.Arg268Cys | None reported | gnomAD |
| p.Arg268His | None reported | gnomAD |
| p.Val272Ala | None reported | gnomAD |
| p.Ile273Val | None reported | gnomAD |
| p.Ala275Thr | None reported | gnomAD |
| p.Ala282Gly | None reported | gnomAD |
| p.Ser284Gly | None reported | gnomAD |
| p.Val287Ile | Hereditary spastic paraplegia 13 | ClinVar |
| p.Leu291Val | Spastic Paraplegia | (Bross and Fernandez-Guerra, 2016)  ClinVar  gnomAD  NHBLI ESP |
| p.Lys292Asn | None reported | gnomAD |
| p.Val293Ile | None reported | gnomAD |
| p.Val298Leu | None reported | gnomAD |
| p.Asn308Ser | None reported | gnomAD |
| p.Lys310Arg | None reported | gnomAD |
| p.Met316Ile | None reported | gnomAD |
| p.Met316Thr | None reported | gnomAD |
| p.Met316Val | None reported | gnomAD |
| p.Ile318Met | None reported | gnomAD |
| p.Ile318Val | None reported | gnomAD |
| p.Ala319Val | None reported | gnomAD |
| p.Ala323Val | None reported | gnomAD |
| p.Gly326Ala | None reported | gnomAD  NHBLI ESP |
| p.Glu328Val | None reported | gnomAD |
| p.Glu335Lys | None reported | gnomAD |
| p.Val337Ile | None reported | gnomAD |
| p.Gln338Arg | None reported | gnomAD |
| p.Gln338His | None reported | gnomAD |
| p.His340Tyr | None reported | gnomAD |
| p.Asp341Ala | None reported | gnomAD |
| p.Val345Ala | None reported | gnomAD |
| p.Gly346Ala | None reported | gnomAD |
| p.Ile349Thr | None reported | gnomAD |
| p.Asp354Asn | None reported | gnomAD |
| p.Asp354Val | None reported | gnomAD |
| p.Met356Ile | None reported | gnomAD |
| p.Met356Val | None reported | gnomAD |
| p.Leu357Phe | None reported | gnomAD |
| p.Asp363Glu | None reported | gnomAD |
| p.Asp363Gly | None reported | gnomAD |
| p.Lys364Arg | None reported | gnomAD |
| p.Ala365Asp | None reported | gnomAD |
| p.Ala365Val | None reported | gnomAD |
| p.Ile367Thr | None reported | gnomAD |
| p.Lys369Asn | None reported | gnomAD |
| p.Arg370Cys | None reported | gnomAD |
| p.Arg370His | None reported | (Bross and Fernandez-Guerra, 2016)  gnomAD |
| p.Arg370Ser | None reported | gnomAD |
| p.Glu373Ala | None reported | gnomAD |
| p.Asp379Gly | None reported | (Hansen et al., 2007; Bross and Fernandez-Guerra, 2016)  gnomAD |
| p.Thr381Ala | None reported | gnomAD |
| p.Thr382Ile | None reported | gnomAD |
| p.Ser383Asn | None reported | gnomAD |
| p.Glu392Gly | None reported | gnomAD |
| p.Arg393Gln | None reported | gnomAD |
| p.Leu394Phe | None reported | gnomAD |
| p.Leu397Val | None reported | gnomAD |
| p.Val403Met | Hereditary spastic paraplegia 13 | ClinVar  GnomAD |
| p.Gly407Ser | Spastic paraplegia | ClinVar  GnomAD |
| p.Lys418Thr | None reported | gnomAD |
| p.Asp419Glu | None reported | gnomAD  NHBLI ESP |
| p.Leu425Phe | None reported | gnomAD |
| p.Arg429Thr | None reported | gnomAD |
| p.Val432Ile | None reported | gnomAD |
| p.Ile436Val | None reported | gnomAD |
| p.Cys442Arg | Spastic paraplegia | ClinVar |
| p.Cys442Trp | None reported | gnomAD |
| p.Arg446Gln | None reported | gnomAD |
| p.Arg446Gly | None reported | gnomAD |
| p.Ile448Leu | None reported | gnomAD |
| p.Ala450Pro | None reported | gnomAD |
| p.Asp452Asn | None reported | gnomAD |
| p.Ala457Thr | None reported | gnomAD |
| p.Gln461Glu | Autosomal dominant form of spastic paraplegia (SPG13) | (Hansen et al., 2007; Bross and Fernandez-Guerra, 2016) |
| p.Gln461Lys | None reported | gnomAD |
| p.Ile463Thr | Hereditary spastic paraplegia 13 | ClinVar |
| p.Gly464Asp | None reported | gnomAD |
| p.Ile465Leu | None reported | gnomAD |
| p.Ile465Val | Spastic paraplegia | ClinVar  gnomAD |
| p.Lys469Glu | None reported | gnomAD |
| p.Lys473Gln | None reported | gnomAD |
| p.Ala476Ser | None reported | ClinVar  gnomAD  NHBLI ESP |
| p.Ala476Val | None reported | gnomAD |
| p.Met477Val | None reported | gnomAD |
| p.Ile479Leu | None reported | gnomAD |
| p.Ala483Thr | None reported | gnomAD |
| p.Gly484Ser | None reported | gnomAD |
| p.Ser488Thr | None reported | gnomAD |
| p.Leu489Phe | None reported | gnomAD |
| p.Ile490Thr | None reported | gnomAD |
| p.Ile490Val | None reported | gnomAD |
| p.Val491Ala | None reported | gnomAD  NHBLI ESP |
| p.Gln496His | None reported | gnomAD |
| p.Ser497Asn | None reported | gnomAD |
| p.Glu500Lys | None reported | gnomAD |
| p.Val501Ala | None reported | gnomAD |
| p.Asp504Asn | None reported | gnomAD |
| p. Ala505Thr | None reported | gnomAD |
| p.Met506Val | None reported | gnomAD |
| p.Gly508Ala | None reported | gnomAD |
| p.Gly508Arg | None reported | gnomAD |
| p.Val511Leu | None reported | gnomAD |
| p.Thr522Arg | None reported | gnomAD |
| p.Asp531Asn | None reported | gnomAD |
| p.Ala536Ser | None reported | gnomAD |
| p.Ala536Val | Hereditary spastic paraplegia 13 | ClinVar |
| p.Thr540Ala | None reported | gnomAD |
| p.Val544Ala | None reported | gnomAD |
| p.Val544Leu | None reported | gnomAD |
| p.Ile549Met | None reported | gnomAD |
| p.Lys551Arg | None reported | gnomAD |
| p.Glu552Gly | None reported | gnomAD |
| p.Lys554Asn | None reported | gnomAD |
| p.Pro556Ser | None reported | gnomAD |
| p.Met558Arg | None reported | gnomAD |
| p.Gly559Asp | None reported | gnomAD |
| p.Met561Leu | None reported | gnomAD |
| p.Met561Val | Spastic paraplegia | ClinVar  gnomAD  NHBLI ESP |
| p.Gly563Ala | Spastic Paraplegia | (Hansen et al., 2007; Bross and Fernandez-Guerra, 2016)  ClinVar  gnomAD  NHBLI ESP |
| p.Met564Val | None reported | gnomAD |
| p.Met568Thr | None reported | gnomAD |
| p.Met568Val | None reported | gnomAD |
| p.Gly571Ser | None reported | gnomAD |
| p.Gly571Val | Spastic Paraplegia | ClinVar  gnomAD  NHBLI ESP |

^1^ The table lists all the found hHsp60 missense mutations. For each is reported the associated disease, if known, and the database or the literature source in which the genetic variant is reported.
